# Burden of rare genetic variants in genes associated with cancer among Malawian cervical cancer patients

**DOI:** 10.4102/ajlm.v15i1.3073

**Published:** 2026-05-15

**Authors:** Samuel D. Gwayi, Tamiwe Tomoka, Emile R. Chimusa, George Fedoriw, Benjamin Kumwenda

**Affiliations:** 1 Department of Biomedical Sciences, School of Life Science and Allied Health Professions, Kamuzu University of Health Sciences, Blantyre, Malawi; 2 Department of Medicine, University of North Carolina, Lilongwe Project, Lilongwe, Malawi; 3 Department of Human Genetics and Forensic Genetics, Northumbria University, Newcastle, United Kingdom; 4 Department of Food Biotechnology, University of Johannesburg, Johannesburg, South Africa

**Keywords:** cervical cancer, human papillomavirus, whole genome sequencing, qualifying variants, rare variants, Malawian women

## Abstract

**Background:**

Cervical cancer (CC) is one of the most common cancer types affecting women globally. Cervical cancer is largely associated with human papillomavirus infections; however, approximately 5% to 11% of CC cases are non-human papillomavirus virus-related. Malawi has the second highest CC prevalence and mortality rate worldwide.

**Objective:**

This study investigated the burden of rare genetic variants in genes associated with CC among Malawian women.

**Methods:**

Ethical approval was obtained from the National Health Science Research committee on 28 August 2023. Whole-genome sequencing was performed on 20 Malawian CC patients, followed by variant discovery and annotation using the Genome Analysis Toolkit and Ensembl’s Variant Effector Predictor. Test for Rare Variants Against Public Database was performed on qualifying variants using 76 156 genomes from the Genome Aggregation database as controls. Bonferroni correction was applied to account for multiple testing.

**Results:**

We identified 372 genes with a significant burden of rare variants (*p* < 0.05), including *FAT1* (*p* = 0.0003), a known tumour suppressor gene associated with CC. After Bonferroni correction (*p* < 6.7×10^−05^), significance shifted to *HTR3B, EPHA8, ABCC2*, and *SEC23B* genes linked to various cancer types.

**Conclusion:**

A significant burden of rare variants associated with CC was observed in known genes associated with lung, ovarian and osteosarcoma cancers, suggesting an increased population risk of developing CC and other cancers among Malawian women that needs further investigation.

**What this study adds:**

The high burden of rare variants in the *FAT1* gene, and a significant rare–variant burden in other cancer associated genes, supports a broader CC genetic risk and possible novel roles requiring further exploration.

## Introduction

Cervical cancer (CC) affects cells of the lower part of the uterus, called the cervix.^1^ The cancer occurs because of the accelerated growth of cervical cells and resistance to programmed cell death, resulting in the formation and accumulation of abnormal cells.^2^ These are cancerous cells that invade neighbouring tissues and spread through metastasis.

Cervical cancer is a global problem, as it is one of the leading causes of death among women globally.^3^ It is estimated that 500 000 cases and over 300 000 deaths occurred globally because of CC in 2018, with an incidence rate of 1.31 per 10 000 women.^4^ It is the second most common cause of death among women in developing countries.^5^ Cervical cancer (CC) patients experience local disease progression, causing fistulas, pain, urethral obstruction, and significant suffering, culminating in death.^6^ There are geographical variations in the distribution of CC globally, with the western Asia and Australia-New Zealand regions having as little as 1.0 case per 10 000 women. The African continent was found to have a very high CC burden, with an average age-standardised incidence rate (ASIR) of between 1.5 and 4.2 cases per 10 000 women, with the exception of North Africa. This was also observed in Eastern Europe, Southeastern Asia, Southern America, and the Caribbean, which also had a world-age standardised rate of about 1.5 cases per 10 000 women.^7^

Within Africa, sub-Saharan countries are severely affected by CC, as indicated by the average ASIR.^8^ The region has an overall average ASIR of 3.49 per 10 000 women. However, the incidence rate varies greatly within this region, with reported rates of 4.34 per 10 000 women in Southern Africa, 4.01 per 10 000 in Eastern Africa, 2.96 per 10 000 in Western Africa, and 2.68 per 10 000 in Central Africa.^9^

Malawi, a sub-Saharan country, has the second highest CC mortality rate and ASIR, after Eswatini, which has the highest global CC mortality rate. Globally, the mortality rate and ASIR are 7.3 per 100 000 per year and 13.3 per 100 000 per year. Malawi has a CC mortality rate and ASIR of 51.5 per 100 000 per year and 67.9 per 100 000 per year. The high prevalence of HIV and human papillomavirus infections have been attributed to the high incidence of CC in the country.^10^ Thus, there have been efforts by the Malawi government through the Ministry of Health (MoH) to combat CC in the country. Programmes promoting CC screening as well as vaccination against human papillomavirus as part of disease control have been implemented in various health care centres.^11^ However, despite these efforts, the incidence of CC continues to increase, and the treatment of invasive cancer is associated with poor prognosis. While high-risk HPVs are largely associated with CC, host genetic susceptibility plays another major role in disease development.^12^ Many genetic-based studies conducted in different populations to elucidate the genetic markers associated with CC have identified single nucleotide polymorphisms and variants.^13,14,15,16^ These studies revealed that, in addition to human papillomavirus infections, genetic variants contribute to the disease. Genetic variants are broadly classified as common or rare based on their minor allele frequency (MAF) in a given population. Rare genetic variants, defined as variants with a MAF of at most 10% (MAF ≤ 0.1), which are usually not detected during genome-wide association studies, and mutations in genes such as *BRCA1, BRCA2, TP53, ATM, CHEK2, BRIP1*, and *PALB2*, have also been reported to play a significant role in cancer predisposition.^17,18,19,20^ The rare variants are evaluated via a burden association test, which assesses their collective impact on diseases that are complex in nature.^21^

Despite the high CC incidence and mortality rate in Malawi, rare genetic markers that may predispose women to CC have not been investigated. Thus, this study investigated the burden of rare genetic variants in genes associated with CC among Malawian women.

## Methods

### Ethical considerations

Prior to participation, consent was obtained from each participant via a written informed consent form. The participants were assigned study identification numbers, and no participant name was used during sample processing and data analysis. Data analysis was performed on a secured bioinformatics server with access granted only to the main investigator. Prior to conducting the study, the study protocol was submitted to Malawi National Health Science Research Committee for ethical review. The National Health Science Research Committee approved the study on 28 August 2023, with the approval number 4048. Sample collection, processing, and whole–genome sequencing were carried out from 01 October 2023 to 19 July 2024.

### Sample collection and sequencing

This study used a case–control association test design utilising whole-genome sequences from 20 Malawian women diagnosed with CC as cases, and 76 156 genomes from the Genome Aggregation Database as controls.^22^ This was a pilot study, and given the limited sample size, we used a rare variant burden test, which is appropriate because it aggregates multiple rare variants within a gene or genomic region into a single test statistic rather than testing each variant individually, thereby enhancing the ability to detect collective effects that single–variant tests would be underpowered to find.^23^ The patients’ average age was 49.05 years, with a minimum age of 31 years and maximum age of 64 years. The clinical characteristics of the study cohort included HIV status, with four out of 20 participants (20%) testing HIV positive and the remaining 16 (80%) being HIV negative at the time of diagnosis. All participants were newly diagnosed with CC, and aside from HIV infection, there were no documented comorbidities in the clinical records. The study site was the Malawi National Cancer Center, in Lilongwe, Malawi. Participants were enrolled in the study on the basis of their confirmed diagnosis of CC.

After obtaining consent to conduct the study, we collected whole blood samples from the patients between 01 October and 30 November 2023. DNA extraction was performed using the Qiagen Max Kit (QIAGEN GmbH, QIAGEN Str. 1, D-40724 Hilden, Germany).^24^ The whole-genome sequencing with an average reads depth of 30X, was done at the Centre for Genomics Research (CGR) in the United Kingdom using the NovaSeq X Plus Illumina platform (Illumina, Inc., San Diego, California, USA) in June 2024.

### Data management and bioinformatics analysis

Whole-genome sequence reads for the 20 samples were obtained from the Centre for Genomics Research server in FASTQ format and stored on a secure local server running Ubuntu 18.04.5 LTS (Canonical Ltd, London, UK) for analysis in July 2024. Data trimming was performed using cutadapt version 4.5Q (Marcel Martin, National Bioinformatics Infrastructure Sweden) with the option -O 3 to ensure that trimming of adapter sequences was done at the 3’ end, matching 3 base pairs (bp) or more. Low-quality bases were removed using a minimum window quality score of 20. Reads shorter than 15 bp were also removed, followed by quality assessment using FastQC and MultiQC.^25,26^ All of the sample sequences that passed the per sequence quality score, mini quality score and per base N content, and GC content, and also passed the duplication and adapter contamination level quality checks, were included for further analysis. Assembly and variant discovery were performed using Burrows–Wheeler Aligner and Genome Analysis Toolkit following best practice guidelines.^27^ The reads were aligned to the Genome Reference Consortium Human Genome Building 38 parch 14 via the Burrows–Wheeler Aligner.^28^ Variant calling was performed on recalibrated bases via Genome Analysis Toolkit Haplotype Caller, followed by joint genotype calling on the individual sample generating a genotype Variant Call Format file. The combined genotype Variant Call Format file contained raw variants for all 20 samples. Single nucleotide polymorphisms were separated from Insertion and Deletions, creating two separate files. These were then filtered using Genome Analysis Toolkit hard-filter before being combined again into one file containing an analysis-ready Variant Call Format file.

### Variant annotation and filtering

A server-based Ensembl Variant Effector Predictor version 108 (EMBL-EBI [European Bioinformatics Institute]). was downloaded onto the server and the variants were annotated based on their genomic location and impact using Variant Effector Predictor.^29^ Variants in coding regions were selected and reannotated for their predicted impact using Sorting Intolerant from Tolerant and Polymorphism Phenotyping function predictors, and their frequency in the 1000 Genomes Project database and Genome Aggregation Database In this study, all variants with a MAF of less than or equal to 0.1 were classified as rare variants.^30^

On the basis of this process, all deleterious, probably or possibly damaging variants with MAF of ≤ 0.1 in the Genome Aggregation Database and the 1000 Genomes Project database were classified as qualifying variants and selected for burden association testing.

### Burden association test and gene annotation

Our analysis leveraged public control genome data rather than matched individual–level genotype data; as such, we employed Test for Rare variants Against Public Database for rare variant burden testing. This association test is based on comparing the burden of rare variants in genes between cases and public controls using summary statistics, which is not feasible with methods such as Sequence Kernel Association Test that require individual–level genotypes and covariate information.^31^ Thus, a single nucleotide polymorphism file was created based on the qualifying variants, and genotypes in cases and controls were obtained using the single nucleotide polymorphism file. A one-sided Fisher’s exact test was performed using the default Test for Rare variants Against Public Database script called *Burden R*.^31^ A *p*-value threshold of less than 0.05 was initially used, followed by Bonferroni correction to account for multiple testing.^32^ This generated a corrected *p*-value of 6.7 × 10^−05^. Genes with significant burden of rare variants were annotated via the Database for Annotation, Visualization and Integrated Discovery tool for disease association and pathway analysis.^33^

## Results

There were 17 443 881 variants called from the 20 Malawian CC cases using Genome Analysis Toolkit. Among these, 9206 variants were classified as high or moderate impact variants present in coding regions of the genomes. Following further filtering of the variants based on Sorting Intolerant from Tolerant, Polymorphism Phenotype v2 (SIFT is developed by researchers at the Fred Hutchinson Cancer Research Center (FHCRC) and the J. Craig Venter Institute while Polyphen-v2 is developed and maintained by the Sunyaev Lab at Brigham and Women’s Hospital, Harvard Medical School) and MAF criteria, 1173 variants were classified as qualifying variants for the association test. These variants were detected in 740 genes among the CC patients. Following the burden association test, 372 genes had an initial significant burden of rare variants (*p* ≤ 0.05). Bonferroni correction for multiple testing resulted in 51 genes with a significant burden of rare variants on the basis of the adjusted *p*-value (*p* < 6.7 × 10^−05^).

**FIGURE 1:**
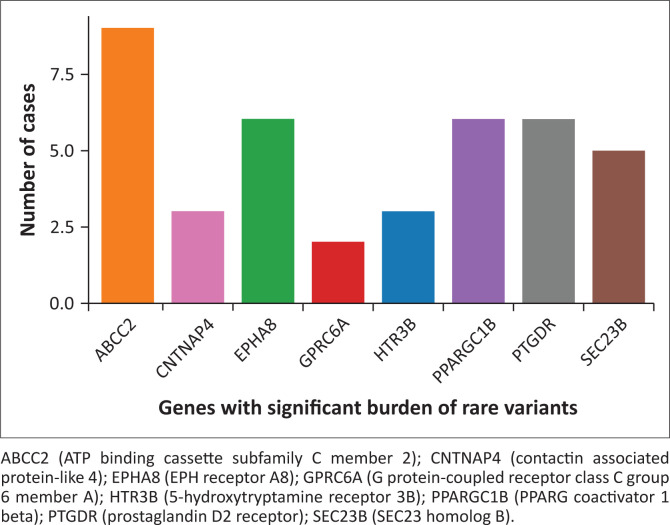
Known genes associated with cancer carrying a significant burden of rare variants among Malawian cervical cancer cases.

Among the genes with initial significance burden was the *FAT1* gene (*p* = 0.0003), whereas after applying the Bonferroni correction and annotation of genes with significant burden using the Database for Annotation, Visualization and Integrated Discovery tool, the significant burden of rare variants was observed in 35 genes associated with human diseases, including eight genes, *HTR3B, EPHA8, ABCC2* and *SEC23B*, which were associated with different cancer types on the basis of Database for Annotation, Visualization and Integrated Discovery annotation (Figure 1 and Table 1).

**TABLE 1:** List of genes with a high burden of rare variants associated with cancer.

Gene name	Gene description	Previously associated cancer type	TRAPD dominant model *p*-values	Association with cervical cancer among Malawian women
*ABCC2*	ATP binding cassette subfamily C member 2	Ovarian	3.52 × 10^−07^	Significant
*CNTNAP4*	contactin associated protein family member 4	Osteosarcoma	2.21 × 10^−05^	Significant
*EPHA8*	EPH receptor A8	Epithelial ovarian	2.42 × 10^−05^	Significant
*FAT1*	FAT atypical cadherin 1	Cervical cancer	3.67 × 10^−04^	Non-significant
*GPRC6A*	G protein-coupled receptor class C group 6 member A	Prostate	3.28 × 10^−06^	Significant
*HTR3B*	5-hydroxytryptamine receptor 3B	Lung	3.28 × 10^−06^	Significant
*PPARGC1B*	PPARG coactivator 1 beta	Pan-cancer	4.97 × 10^−05^	Significant
*PTGDR*	prostaglandin D2 receptor	Prostanoid signalling in cancer	3.32 × 10^−06^	Significant
*SEC23B*	SEC23 homolog B, COPII coat complex component	Noncanonical role in cancer	5.14 × 10^−05^	Significant

TRAPD, Test for Rare variants Against Public Database; DAVID, Database for Annotation, Visualization and Integrated Discovery.

TRAPD dominant model *p*-values, associated conditions, cancer type based on DAVID annotation and observed association with cervical cancer are presented.

**FIGURE 2:**
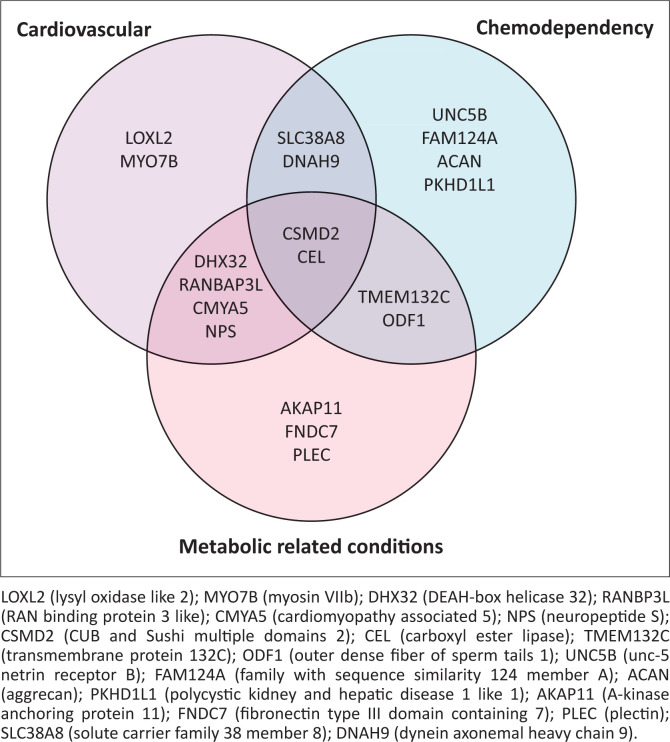
Genes with a significant burden of rare variants associated with major conditions in Malawian cervical cancer cases, including cardiovascular, metabolic and chemodependency-related conditions, as well as shared genes accross these conditions, based on DAVID annotation.

A significant burden of rare variants was also observed in 27 genes associated with various human conditions. Most of these genes were associated with cardiovascular disease, as well as chemodependency and metabolism related abnormalities, based on Database for Annotation, Visualization and Integrated Discovery annotation (Figure 2).

## Discussion

This study investigated the burden of rare genetic variants (MAF < 0.1 in the 1000 Genome Project and Genome Aggregation Database), in genes associated with CC among Malawian women. To our knowledge, this is the first study conducted among Malawian women to investigate CC genetic markers, and the results provide significant insights into the genetic nature of CC among Malawian women.

The findings of this work highlight the notable burden of rare variants with a significant *p*-value of 0.0003 in the tumour suppressor *FAT1* gene. The gene controls cell respiration and signals for cell–cell contact when the physiological conditions are normal within the body. Our findings suggest that the *FAT1* gene may have a potential role in CC causation among Malawian women, as mutations in the gene have been linked to cervical, colorectal and breast, as well as head and neck cancers.^34^ Suppressive mutations in the *FAT1* gene are associated with type changes in epithelial cells and cancer cell initiation.^35^ However, in our study, the association between *FAT1* gene and CC was not statistically significant.

After applying the Bonferroni correction (adjusted *p*-value, 6.7 × 10^−05^), we identified a significant burden of rare variants in several genes, including the *HTR3B, EPHA8* and *ABCC2* genes. These are known genes associated with different cancer types, such as ovarian, lung, and breast cancers. The *HTR3B* gene, for example, because of its role in neurotransmitter signalling, has been associated with worse clinical manifestations among patients during cancer therapy, including nausea and vomiting.^36^ Alterations in genes have been described as risk signatures for breast cancer and are associated with shorter overall survival in patients with breast and lung cancers.^37,38^ The high burden of rare variants in this gene suggests that the identified gene plays a significant role in CC causation among Malawian women. On the other hand, the *EPHA8* gene is known to play an important role in the formation of new vessels and cancer metastasis during tumour formation. The gene has been classified as a cancer-causing agent because of its role in ovarian cancer causation and progression.^39^ Futhermore, the *ABCC2* gene plays a critical role in lung cancer treatment outcomes, as it has been associated with poor response to chemotherapy and overall survival in patients with lung cancer.^40^ This gene has been identified as a possible candidate for lung cancer therapy.^41^ Thus, our study suggests that both *EPHA8* and *ABCC2* genes also play an important role CC development among Malawian women.

Other cancer-related genes with a significant burden of rare variants identified in this study included *SEC23B, GPRC6A, PPARGC1B, CNTNAP4*, and *PTGDR* genes. While higher expression of the *SEC23B* gene has been associated with highly metastatic breast cancer, variations in the gene have been classified as having a yet to be agreed upon role in cancer progression.^42,43^ On the other hand, *GPRC6A* gene overexpression is associated mostly with male prostate cancer.^44,45^ Apart from these genes, a recent study among the Chinese population revealed that variations in the *PPARGC1B* gene are associated with an increased risk of gastric cancer in both sexes.^46^ However, as early as 2006, inherited single nucleotide polymorphisms in the *PPARGC1B* gene were associated with breast cancers within families.^47^ A correlation between variations in the gene and breast cancer was also reported in 2011, when it was discovered that individuals with some polymorphisms in the *PPARGC1B* gene were at increased risk of breast cancer.^48^ Furthermore, regarding variations in the *CNTNAP4* and *PTGDR* genes, studies have shown that mutations in *CNTNAP4* and *PTGDR* genes are linked to osteosarcoma and colorectal cancer.^49,50^ Therefore, while the four genes have not been previously associated with CC, our findings suggest their role in CC disease among Malawian women.

Given that cancer is classified as a complex disease, the discovery of 27 additional genes identified as having a significant burden of rare variants and associated with conditions not directly related to cancer emphasises the diversity of biological pathways involved and the complexity of the interactions among these genetic factors. These interactions may indicate a role of the identified genetic factors in CC oncogenesis among Malawian women, and require further investigation.

### Limitations

In this study, we utilised public genomes as controls rather than locally collected healthy Malawian women controls. This could introduce bias resulting from differences in genetic background or population structure. Future studies should include matched local controls to confirm and extend our findings. We were also unable to apply individual–level rare variant tests such as Sequence Kernel Association Test because of smaller sample size and lack of matched control data. Sequence Kernel Association Test requires individual–level genotype data, which is not available when using summary–level external control datasets. This study is based on genetic association data without experimental or functional validation of the rare variants identified in genes. Thus, the biological effects and mechanisms of the rare variants remain unclear. Future work should involve laboratory-based (in vitro or in vivo) studies to confirm the roles of the identified rare variants.

### Conclusion

This study revealed that while a high burden of rare variants was present in a known gene associated with CC (*FAT1)* among Malawian women, this was not statistically supported. However, the study revealed that *HTR3B, EPHA8, ABCC2, SEC23B, GPRC6A, PPARGC1B, CNTNAP4*, and *PTGDR* genes, previously associated with other cancer types, had a significantly high burden of rare variants in CC cases compared with controls among Malawian women. The findings suggest a potential role of the identified genes in CC causation and shared cancer susceptibility among Malawian CC patients. However, future studies should focus on the functional roles and clinical implications of these genes in CC disease.
